# A Rare Case of Lambert-Eaton Myasthenia Syndrome With Dysphasia and Dysarthria

**DOI:** 10.7759/cureus.86700

**Published:** 2025-06-24

**Authors:** Hridya Harimohan, Mia Yasonova, Marah Sukkar, Katayoun Sabetian

**Affiliations:** 1 Internal Medicine, Kern Medical, Bakersfield, USA; 2 Neurology, Kern Medical, Bakersfield, USA

**Keywords:** antibody, dysarthria, dysphagia, lambert-eaton myasthenic syndrome, plasmapharesis

## Abstract

Lambert-Eaton Myasthenic Syndrome (LEMS) is a rare autoimmune disorder resulting in muscle weakness. LEMS typically presents with progressive proximal muscle weakness, especially in the lower extremities, areflexia, and autonomic dysfunction. Bulbar involvement and respiratory compromise tend to occur later in the disease course and usually in severe, advanced cases. We report a unique case of a 50-year-old male with a known history of LEMS, autoimmune retinopathy, erectile dysfunction, and prediabetes who presented with progressive bulbar symptoms - slurred speech and difficulty swallowing - without accompanying weakness in the upper or lower extremities. Despite receiving four doses of intravenous immunoglobulin (IVIG) prior to admission with no improvement, he showed a significant clinical response to six sessions of plasmapheresis. Notably, the absence of limb weakness and preserved motor strength throughout his hospital stay raised diagnostic uncertainty, prompting evaluation for myasthenia gravis, which was ruled out through negative acetylcholine receptor antibody testing and reaffirmed voltage-gated calcium channel (VGCC) antibody positivity. Chest imaging showed no underlying malignancy. This case illustrates an atypical LEMS presentation dominated by severe bulbar dysfunction in the absence of limb involvement or respiratory failure, which is uncommon and may mimic other neuromuscular junction disorders such as myasthenia gravis. The favorable response to plasmapheresis emphasizes the importance of early recognition and aggressive immunomodulatory treatment in preventing clinical deterioration. Clinicians should maintain a high index of suspicion for LEMS even in unusual presentations to ensure timely and appropriate management.

## Introduction

Lambert-Eaton Myasthenic Syndrome (LEMS) is an autoimmune disorder that affects the neuromuscular junction, where autoantibodies target presynaptic voltage-gated calcium channels (VGCC) [1]. The binding of these autoantibodies reduces calcium influx into the presynaptic nerve terminal, leading to decreased acetylcholine release and resulting in muscle weakness [2]. The estimated incidence of LEMS is 0.48 per million [3]. Approximately 50-60% of LEMS cases are paraneoplastic, most commonly associated with small-cell lung carcinoma, which expresses functional VGCCs, while the remaining cases are non-paraneoplastic [4]. LEMS typically presents with proximal muscle weakness, autonomic dysfunction, and areflexia [5]. Severe bulbar muscle involvement can lead to respiratory failure, presenting a diagnostic challenge, particularly in differentiating it from conditions like myasthenia gravis. However, bulbar involvement usually occurs in the later stages of chronic, progressive LEMS. Here, we present an atypical case of LEMS with severe bulbar weakness but normal upper and lower extremity strength. This case is rare because even after a thorough literature search, we could not find a similar case of LEMS presenting with dysphagia without weakness in the extremities.

## Case presentation

A 50-year-old male with a past medical history of LEMS on amifampridine phosphate, autoimmune retinopathy on methotrexate with folic acid supplementation, erectile dysfunction, and prediabetes presented to the emergency department with slurred speech and difficulty swallowing over the past three weeks. He states that he eats very slowly, takes multiple attempts to swallow, and is only able to eat soft foods with difficulty, along with liquids. His symptoms had been gradually worsening, and he had recently been admitted to another hospital for the same symptoms, where he underwent a detailed workup, which was negative. Despite receiving four doses of intravenous immunoglobulin (IVIG), there was no significant improvement, and he was referred to our hospital for further evaluation. He denied any recent infections or use of new medications that could have triggered his condition. 

The patient had been diagnosed with LEMS 10 years ago and was found to be positive for voltage-gated calcium channel antibodies at that time. He underwent thymectomy, which revealed thymic hyperplasia, and had been maintained on amifampridine phosphate 10 mg daily, which he was compliant with, and had not experienced any recent dosage changes. Additionally, he had been diagnosed with autoimmune retinopathy and was being treated with methotrexate (2.5 mg, eight tablets weekly) with supplemental folic acid, but was legally blind. 

Upon presentation to the emergency department at our hospital, his vital signs were significant for tachypnea with a respiratory rate of 39 breaths per minute, elevated blood pressure of 145/106 mmHg, and a heart rate of 90 beats per minute, with oxygen saturation of 97% in room air. Physical examination was notable for dysarthria, difficulty swallowing requiring frequent suctioning during the evaluation, and dysphonia. He was not in significant respiratory distress, and on auscultation, his breath sounds were clear with no added sounds. Neurological examination revealed severe weakness of the tongue, absent reflexes bilaterally in both upper and lower extremities, and chronic decreased visual acuity. The rest of the physical exam was unremarkable, with negative Babinski signs, no ptosis, normal extraocular movements, normal strength in both upper and lower extremities, and intact sensation to pinprick and light touch in both upper and lower extremities (Table 1).

**Table 1:** Laboratory results

Lab test	Value	Reference range
Acetylcholine receptor binding antibody	<0.30 nmol/L	≤0.30 nmol/L - normal; 0.31-0.49 nmol/L - equivocal; 0.50 nmol/L - positive
Acetylcholine receptor blocking antibody	<15	<15% inhibition
Acetylcholine receptor modulating antibody	<1	<1 % inhibition
Voltage-gated calcium channel (VGCC) type P/Q antibody	>30	<30 pmol/L
VGCC type N Ab	59	<54 pmol/L

Speech language pathology was evaluated, and the modified barium swallow study showed decreased epiglottic inversion and decreased pharyngeal constriction. No aspiration, but deep penetration was noted, which diminished with chin tuck. Vallecular/pharyngeal residue was noted and needed multiple swallows to clear (more with liquids than solids).

Following neurological consultation, he underwent six sessions of plasmapheresis, and during his hospital course, he was continued on amifampridine phosphate 10 mg orally daily. We also refrained from using any medications that could potentially trigger or exacerbate his condition. After plasmapheresis, there was significant improvement in both dysphagia and dysarthria, while his chronic blurry vision remained stable. Given the atypical presentation of severe bulbar involvement without significant weakness in the upper and lower extremities, myasthenia gravis was considered a close differential diagnosis. As such, we repeated laboratory tests, including the myasthenia gravis panel, which included blocking and modulating acetylcholine receptor antibodies (both negative) and voltage-gated calcium channel antibodies (which were positive). His motor strength in both upper and lower extremities remained intact throughout the hospital course, and there was no evidence of respiratory or hemodynamic decompensation.

## Discussion

Our patient exhibited some of the classic features of LEMS, a rare autoimmune disorder, including loss of deep tendon reflexes and autonomic dysfunction, as evidenced by his erectile dysfunction. However, in the typical course of LEMS, muscle weakness often begins in the lower extremities, gradually progresses to the oculobulbar muscles, and, in severe cases, affects respiratory muscles [6]. In contrast, our patient presented with severe bulbar weakness, which caused significant dysphagia, dysarthria, and dysphonia. Notably, his motor strength in both the upper and lower extremities remained intact at the time of presentation and throughout the hospital course. This atypical presentation led to diagnostic challenges, particularly as other neuromuscular disorders, such as myasthenia gravis, can present similarly with bulbar weakness [7]. Despite repeating the diagnostic workup, the detailed myasthenia gravis panel, which included tests for blocking and modulating acetylcholine receptor antibodies, returned negative results. However, the voltage-gated calcium channel antibodies were positive, which ultimately confirmed the diagnosis of LEMS.

The patient also had a thymectomy in the past, which was negative for thymoma, a finding typically seen in myasthenia gravis, and instead revealed thymic hyperplasia. Another rare but important differential diagnosis to consider was botulism, which shares similarities with LEMS in terms of presentation [8]. However, it is crucial to differentiate between the two conditions, as the management strategies differ significantly. After reviewing the literature, we found very few cases of LEMS presenting with respiratory failure, which further highlights the rarity of such a presentation [9]. Despite the severity of his bulbar weakness, our patient did not experience respiratory distress, and with the prompt initiation of IVIG and plasmapheresis, we were able to prevent further complications in a timely manner. While the majority of LEMS cases are associated with an underlying malignancy, especially small cell lung carcinoma, our patient presented with the rarer paraneoplastic form of LEMS. This distinction underscores the complexity and variability of LEMS presentations, as well as the importance of maintaining a high index of suspicion for accurate diagnosis and appropriate treatment [10].

Since our patient was being followed by an outside facility, we did not have prior or subsequent titers; however, the voltage-gated calcium channel antibody is mainly used for diagnosis and not for correlation with severity [11].

We know that in myasthenia gravis, 15% of patients may present with dysphagia as the only symptom. The mechanism behind the difficulty swallowing in myasthenia gravis may be due to incoordination between the suprahyoid laryngeal elevator muscles and the cricopharyngeal muscles of the upper esophageal sphincter [12]. Since this is the first reported case of dysphagia as the only symptom of LEMS, we infer it is due to the same mechanism.

## Conclusions

The diagnosis of LEMS demands a high level of clinical suspicion, particularly due to the rarity of the condition and the possibility of atypical presentations, as was the case with our patient. LEMS often presents in ways that can easily be mistaken for other, more common neuromuscular disorders, which can lead to delays in diagnosis. This makes it even more crucial to recognize the condition early and begin appropriate treatment promptly, as there is a significant risk of the condition worsening if not addressed in a timely manner. Without intervention, the bulbar weakness seen in LEMS can rapidly progress, potentially leading to life-threatening complications such as respiratory failure, which can ultimately result in death.
